# Book Review: Consolidation of Quantitative and Qualitative Research

**DOI:** 10.3389/fpubh.2018.00207

**Published:** 2018-08-15

**Authors:** Marina Luketina Sunjka

**Affiliations:** European Center for Peace and Development, University for Peace established by the United Nations, Belgrade, Serbia

**Keywords:** qualitative research, quantitative research, multimethod research, study design, methodology

“Consolidation of quantitative and qualitative research” is a book written by the author Prof. Dr. Zivan Ristic. The book was published in 2017 by ECPD, United University for Peace established by the United Nations, with 500 copies printed in Belgrade, Republic of Serbia. The original book review published along with the book, was written by Prof. Dr. Vlastimir Matejic and Prof. Dr. Miodrag Ivanovic.

The book has three chapters. In the first chapter, which relates to the review of basic paradigmatic approaches to research, Ristic elaborates on the so-called “wars of paradigms” (1) which started in the 1970s and spread out into the 1990s. The 2000s were characterized by the need to replace old paradigms with new ones, using a new approach of mixed, qualitative and quantitative research, methods which are elaborated upon in the third chapter of the book. Ristic introduces the importance of the term “paradigm” and talks about realism and relativism in science.

In the second chapter he elaborates on different approaches in science research of undertaking qualitative and quantitative research by observing from the aspect of the paradigmatic approach and from the aspect of the research itself. He gives clear instructions regarding the aim and the goal, the research question, the strategy, the type and the design of either the qualitative or quantitative research, proper sampling, and proper data collection, depending on the type of the research. Finally it is shown how best to analyze it again, depending on the research. Ristic conveys an interesting conclusion where he says that when using a qualitative research method the hypothesis doesn't have to be defined, whereas the definition of the hypothesis is one of the most important segments within quantitative research. Depending on this, as Ristic says in his book, the data collection type will determine whether the research will be qualitative or quantitative. If we collect data in the form of figures aiming to research the links between the variables, or to understand causal relationships between the researched occurrences, we should apply quantitative research. On the other hand, if we are dealing with descriptive data obtained in a natural environment by extensive observation or by using interviews, we should apply qualitative research.

In the third chapter, titled “Third methodological movement,” besides the phrase of the term “research by using mixed methods,” he takes us through all phases of process, from the research question, to design, sampling, data collection and analyses of mixed methods research. Various terms are used to describe the “third methodological movement” such as “integrative research” (2) and “Research by multimethod research” (3).

This book could become an important practical tool for any doctor. In full, by showcasing a number of examples, it explains to future doctors the methodological approach while analyzing, researching, observing, studying and is to be considered as a great help while creating one's own projects, masters thesis or doctorial dissertation. The book by Zivan Ristic also introduces the mixed method research which was later endorsed by Onwuegbuzie (4) who was of the opinion that this was the method of future. A special dimension of the book is presented in a form of research with mixed methods, which (4) supported by saying that time of such type of research is only coming. As per International Journal of Multiple Research Approaches (5), Leech and Onwuegbuzie, claim that mixed research in fact is the most comprehensive approach.

A reader is able to find any answer, regardless of whether he or she is looking for guidance for their masters thesis or doctorial dissertation. This book by Ristic provides answers to questions related to how to formulate a focus of the research, and how to define goals while describing the type of research or while deciding on the type of strategy (quantitative, qualitative, or mixed research).

Proper guidance is of utmost importance when designing a study. Design represents the structure that groups all elements of the study, in order to receive the results which are general, error free and trustable, as said by Dannels (6). Ristic gives instructions on quantitative research design types, such as experimental, correlation, ex post facto, survey, meta-analyses, as well as types of design of qualitative research such as phenomenological, ethnographic, historical, case study qualitative meta-analyses and other, as well as the design of the mixed study. In relation to mixed method research, a number of scientists provided different proposals of study design typologies [(7), pp. 814–815]. The third chapter of the book reveals all design types of mixed study methods including typology by Creswell and Piano Clark, typology by Tashakkori and Newman, typology by Leech and Onwuegbuzie, and then typology by Morgan, then, Morse, typology by Greene (8). Based on the design type, sampling, schemes, and figures will differ. Thanks to the book “Consolidation of quantitative and qualitative research” by Ristic it is possible to find all of the following in one place—types of researches, whether it is quantitative or qualitative, design type, all models of sampling, all models of data collection, as well as the models of interpretations of those important to readers. The visual presentation of the book content related to random data listing could be improved.

This book, published by ECPD of the United Nations university represents a “spelling book” for Ph.D. students preparing their thesis regardless of their scientific field. Being filled with a number of detailed explanations for an each phase of the study, this book is surely on its way to becoing an obligatory literature element while preparing a Ph.D. dissertation.

## Author contributions

The aim of the author of this review was to explore the available literature with same or similar subject related to quantitative and qualitative research and create one review for the book that showcased the subject at its best. After having read a number of similar books covering the theory and practice of consolidating a Ph.D. methodological study, the book of professor doctor Zivan Ristic with its original title Objedinjavanje kvantitativnih i kvalitativnih istrazivanja has stand out the most and the attached book review relates to this particular book. ML is the only author of this this particular book review.

### Conflict of interest statement

The author declares that the research was conducted in the absence of any commercial or financial relationships that could be construed as a potential conflict of interest.
